# Rapid and Effective Lead Elimination Using Cow Manure Derived Biochar: Balance between Inherent Phosphorus Release and Pollutants Immobilization

**DOI:** 10.3390/toxics11010001

**Published:** 2022-12-20

**Authors:** Huabin Wang, Yi Wen, Yu Ding, Zhiqiang Yue, Dan Xu, Ying Liu, Yong Zhang, Rui Xu, Weiqing Zeng

**Affiliations:** 1 School of Energy and Environment Science, Yunnan Normal University, Kunming 650500, China; 2 Yunnan Key Laboratory of Rural Energy Engineering, Kunming 650500, China; 3 Baoshan City Longyang Rural Energy Workstation, Baoshan 678000, China; 4 Yuxi Agricultural Environmental Protection and Rural Energy Workstation, Yuxi 653100, China

**Keywords:** cow manure, biochar, lead pollution, phosphorus release, crop growth

## Abstract

Cow manure derived biochar (CMBC) can serve as a promising functional material, and CMBC can be regarded as an ecofriendly approach compared to conventional ones. CM bioadsorbent can be employed for heavy metal immobilization (such as for lead) as well as an amendment to increase soil fertility (e.g., phosphorus). Few studies have examined the surface interactions between pollutants and bioadsorbents when inherent nutrient release is present. In this work, CMBC was prepared and applied for Pb(II) removal, and the vital roles of released phosphorus from CMBC were comprehensively disclosed. Furthermore, CMBC could immobilize part of the Pb(II) in soil and promote plant growth. CM400 was an effective adsorbent whose calculated *Q_e_* reached 691.34 mg·g^−1^, and it rapidly adsorbed 98.36 mg·g^−1^ of Pb(II) within 1 min. The adsorption mechanisms of Pb(II) by CMBC include ion exchange, physical adsorption, electrostatic attraction, chemical precipitation, surface complexation, and cation–π bond interaction. Based on the residual phosphorus content and adsorption effect, complexation rather than the chemical precipitation had a greater contribution toward adsorption. Besides, as the concentration of Pb(II) increased, the main adsorption mechanisms likely transformed from chemical precipitation to ion exchange and complexation. CMBC not only had a good effect on Pb(II) removal in the solution, but also immobilized the Pb(II) in soil to restrain plant uptake as well as promote plant growth. The main novelty of this work is providing more insights to the cow manure bio adsorbent on Pb immobilization and phosphorus release. This study is expected to serve as a basis and reference for analyzing the release effects of inherent nutrients and the interfacial behaviors with heavy metals when using CMBC and other nutrient–rich carbon–based fertilizers for pollution control.

## 1. Introduction

In recent decades, the risks posed by heavy metals have garnered widespread global attention owing to the persistence, toxicity, and bioaccumulation of these metals. Among them, lead (Pb) is persistent in the environment and can result in both decreased growth and reproduction in plants and animals, in addition to neurological effects in vertebrates, while the potential harm of these effects has attracted growing concerns [1]. In China, lead smelting operations are mostly centered in the south–central and southwest areas, such as at the Huize Pb–Zn deposit in Yunnan Province [2]. Pb(II) removal methods consist of membrane filtration, electrocoagulation, coagulation, photo-catalytic redox procedures, Fenton oxidation, and γ-ray irradiation [3,4]. In particular, it is widely used as an adsorbent to eliminate Pb(II), as the adsorption process can be easily handled at low-cost [5,6]. Therefore, it is essential to create rapid, efficient, and cost-effective adsorbents. Due to the abundance of functional groups, pore structure, and strong affinity for heavy metals in biochar, biochar has become widespread in Pb(II) removal [7].

Over recent years, the production of cow manure (CM) has increased with the developments of the large-scale and intensive cattle breeding industry in China [8]. CM is a valuable resource for the sustainable development of agriculture [9], and is mainly used as fertilizer, energy, and so on [10]. The contents of lignin and cellulose in CM are considerably higher than those in pig manure and chicken manure, which increases the fermentation time [11]. CM is rich in phosphorus, while the heavy metal content is lower than that of pig manure [12], so it is imperative to use CM with a high value. Researchers have found and confirmed that CM can promote the growth of crops and reduce phosphorus application [13]. However, the inherent phosphorus from CM is water extractable phosphorus, which can easily cause eutrophication [14]. Researchers have found that the slow release of phosphorus can be achieved by pyrolysis [15]. Furthermore, the cow manure derived biochar shows more efficient adsorption of heavy metals than wood-derived biochar, as the PO_4_^3−^ and CO_3_^2−^ contained and functional groups improve the adsorption via complexation and precipitation [16]. Thus, cow manure derived biochar is widely used to adsorb heavy metals [17,18]. Compared with compost, the cow manure derived biochar offers a lower cost, easier preparation, and better adsorption [18]. Modifications such as KH_2_PO_4_ or NaOH can significantly improve adsorption [19,20].

Even though the presence of phosphorus in cow manure derived biochar has been found [21], few studies are available regarding the phosphorus release during heavy metal removal or the effect of phosphorus fertilizers during adsorption. Few studies have analyzed the relationship between inherent phosphorus and Pb(II) adsorption by cow manure derived biochar. Therefore, this work focused on a phosphorus-contained biomass, CM, and used a two-step method to prepare biochar functional materials, named CMBC. In this work, the adsorption effects of CMBC on Pb(II) in solution and soil were investigated. Through the adsorption experiment in solution, the differences in adsorption capacity of three kinds of biochar prepared at different pyrolysis temperatures were compared, and the adsorption mechanism of Pb(II) adsorption by CMBC was analyzed by changing the reaction conditions. By simulating lead-contaminated soil and planting Chinese cabbage in lead-contaminated and pollution-free soil, the growth of Chinese cabbage with or without CMBC was compared. In order to compare and analyze the effect of CMBC on reducing the bioenrichment of Pb(II), the Pb(II) content in the roots, stems, and leaves of Chinese cabbage was detected after maturity. The ability of CMBC to promote crop growth was analyzed by comparing the phosphorus content in three parts of Chinese cabbage. Notably, this study aimed to explore the release mechanism of inherent phosphorus and the interactions between inherent phosphorus and Pb(II). Furthermore, CMBC was applied to lead-contaminated soil to verify whether CMBC can solidify heavy metals and promote crop growth.

## 2. Materials and Methods

### 2.1. Experimental Material

Pb(NO_3_)_2_·3H_2_O, HNO_3_ (65–68%), and NaOH were used, and all chemicals were of analytical grade. Further, CM was collected from the Laohadu Demonstration Ranch in Mengzi City, Yunnan Province, China.

### 2.2. Preparation of CMBC

**Scheme 1:**
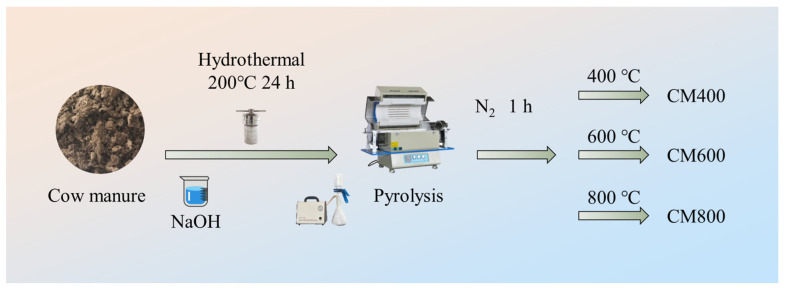
The preparation process for CMBC.

The preparation process for the CMBC is illustrated in Scheme 1. Fresh CM was dried at 85 °C and ground (100-mesh). Firstly, 4 g dried CM, 112 mL deionized water, and 38 mL of NaOH solution (1 mol·L^−1^) were mixed. Secondly, the mixture was shaken with ultrasound for 30 min and stirred for another 2 h. Thirdly, the mixture was hydrothermally heated at 200 °C for 24 h and then rinsed with acetone. Finally, the mixture was dried at 85 °C. CMBC was obtained via pyrolysis under the protection of N_2_ at 400 °C, 600 °C, and 800 °C for 1 h, denoted as CM400, CM600, and CM800, respectively.

### 2.3. Analysis Methods

The elemental composition of biochar was analyzed with elemental analyzer (Elementar Vario EL cube, Langenselbold, Germany). The morphology was analyzed with scanning electron microscopy (SEM) and SEM–EDX at 5 kV (Mira LMS, Tescan, Brno, Czech Republic). The specific surface area (SSA) of biochar was detected by N_2_ adsorption isotherms at 77 K using a Micropore Analyzer (ASAP 2460, Micrometrics, Cumming, GA, USA). The electron binding energy and elemental valence were analyzed using X-ray photoelectron spectroscopy (XPS) (K-Alpha, Thermo Scientific, Waltham, MA, USA). The functional groups were qualitatively examined using a Fourier transform infrared spectrometer (FTIR) (Niolet iN10EA, Thermo Scientific, Waltham, MA, USA). The amount of the elements in the solutions was determined by inductively coupled plasma mass spectrometry (ICP) (Thermo Fisher–X series, Waltham, MA, USA). The contents of the elements in the soil and crop were determined via atomic absorption spectrophotometry (iCETM3500 AAS, Waltham, MA, USA).

### 2.4. Adsorption Experiment

Twenty mg of biochar was weighed, then 20 mL deionized water or Pb(II) solution was added. After oscillation (200 r·min^−1^, 25 °C), 5 mL solution was filtered (0.22 μm) at predetermined times (0.008, 0.083, 0.5, 1, 2, 6, 12, 18, and 24 h), and the phosphorus or Pb contents were determined via ICP. The adsorbent dosage was 1 g·L^−1^, then 20 mL Pb(II) solution was added (50, 100, 200, 300, 400, 500, 600, 800, and 1000 mg·L^−1^), and HNO_3_ and NaOH solutions were used to adjust the pH of solution (pH = 7.0). After 24 h of oscillation (200 r·min^−1^), the sample solution was filtered (0.22 μm), and the Pb(II) content was determined via ICP. When the adsorbent dosage was 1 g·L^−1^ and the initial concentration of Pb(II) was 100 mg·L^−1^, the effects of pH (3.0, 5.0, 7.0, 9.0, and 11.0) and temperature (25 °C, 35 °C, and 45 °C) on the adsorption was explored.

The adsorption capacity of Pb(II) was calculated using Equation (S1) and the experimental results were fitted using the adsorption dynamic model (Equations (S2)–(S5)) and the isothermal adsorption model (Equations (S6) and (S7)). The equations and symbols in the equation are described in the Supplementary Materials (Text S1).

### 2.5. Pot Experiments

Chinese cabbage is a type of common model plant, and it has small, fast growth, growth ability, etc. [22]. Three experimental groups (T_1_: 1 g CM400 application, T_2_: 1 g CM600 application, T_3_: 1 g CM800 application) and two control groups (CK_1_: no pollution treatment, CK_2_: no adsorbent application) were set up in the pot experiment. Firstly, 2 kg screened soil (5 mm) was placed in a plastic pot. Secondly, to simulate the actual conditions of Pb(II) pollution in Yunnan Province, China [23], the experimental groups and CK_2_ were sprayed with Pb(NO_3_)_2_ solution (100 mL, 60 mg·L^−1^), and CK_1_ was sprayed with 100 mL water. We then ripened the loaded soil by pouring a permeable and dried it naturally for 40 days. Thirdly, Chinese cabbage seeds were directly sown into pots (10 seeds per pot), and 1 g CM400, CM600, or CM800 were applied to three experimental groups, respectively, and then covered with 1–2 cm of soil. Each pot was irrigated daily with 150 mL water, and the budding and growth of Chinese cabbage were observed and recorded. Once the Chinese cabbage was mature enough, the Pb(II) and P contents in roots, stems, and leaves were determined.

## 3. Results and Discussion

### 3.1. Characterization

The basic elements of CMBC were shown in Table 1. For CM400, the contents of carbon element (C%), oxygen element (O%), and hydrogen element (H%) were 28.11, 41.48, and 5.05% respectively, while they were 39.21, 37.89, and 1.63% for CM800. Obviously, O% and H% decreased with the increase of pyrolysis temperature, which mainly attributed to the pyrolysis of organic compounds [24]. During carbonization, both H/C and O/C ratios decreased due to the release of functional groups containing H and O [25], thus, the O/C ratios of CM400, CM600, and CM800 were 1.48, 0.07, and 0.04, respectively, and the H/C ratios for them were 0.18, 0.07, and 0.04. Notably, CM400 had the highest O/C and H/C ratios, which indicated that CM400 may have had the most prevalent functional groups and the best adsorption effect on hydrophobic pollutants [26], namely, CM400 may have had a stronger ability to adsorb Pb(II) via complexation [27]. Noteworthy, phosphorus element (P%) was 0.61, 0.34, and 0.27% for CM400, CM600, and CM800, and the results of elemental analysis proved the existence of inherent phosphorus in CMBC.

The SSA is presented in Table 1. The SSA were 2.51, 11.78, and 12.93 m^2^·g^−1^ for CM400, CM600, and CM800, respectively, which showed that the SSA of CM800 was higher than that of CM400 and CM600. In the range of 400–800 ℃, the SSA of CMBC increased with the preparation temperature, and in the range of 400–600 ℃, SSA increased greatly, while in the range of 600–800 °C, SSA increased steadily but slightly. The SSA is associated with physical properties and significantly affects the physical adsorption capacity. Hence, CM800 had better physical adsorption capacity than the two other types of biochar [28]. Moreover, the average pore sizes of CM400, CM600, and CM800 were 837.94, 90.31, and 108.91 Å. The pore size of CM400 is larger than the others, so CM400 may have had a better adsorption effect via pore filling.

**Figure 1:**
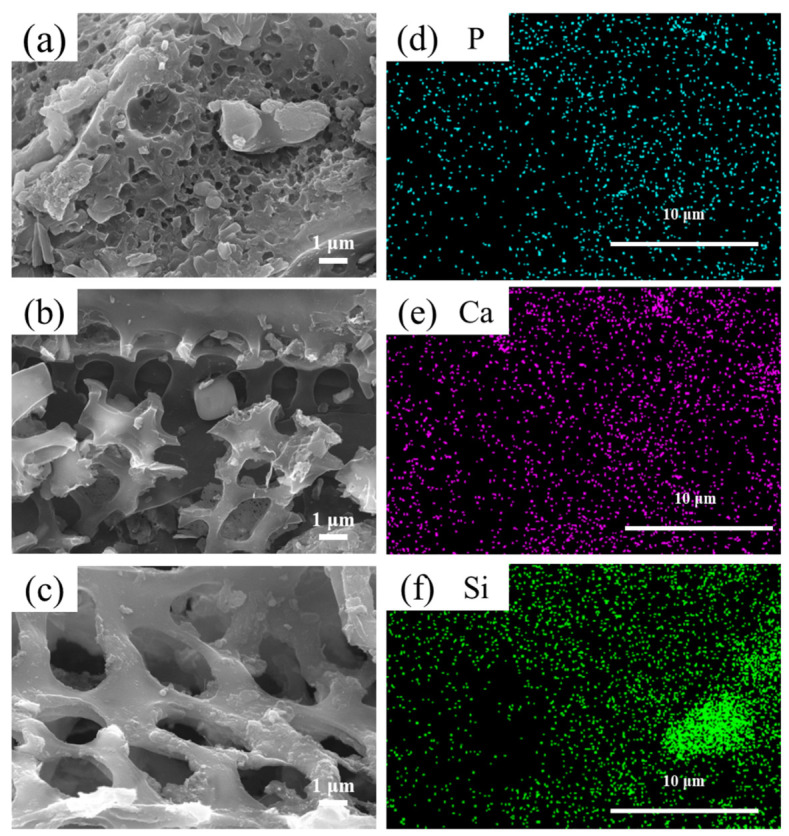
SEM and EDS images of CMBC (**a**–**c**): SEM images of CM400, CM600, and CM800; (**d**–**f**): EDS images for P, Ca, and Si of CM400.

The SEM and SEM-EDS images are shown in Figure 1. The layered porous structure riddled with micropores is presented on CMBC, as CM contains cellulose [29]. The ledge structure of CMBC was related to pyrolysis temperature change; when the pyrolysis temperature ≤600 °C, CM400 and CM600 retained a part of the stereoscopic carbonaceous skeleton structure and bulk accumulation whereas the ledge structure of CM800 became thinner. More large pores were observed on CM800 than CM400 and CM600, thus, the CM800 may have had better physical adsorption capacity [30], which was consistent with the above analysis. Moreover, the CMBC contained Si, Ca, and P.

**Figure 2:**
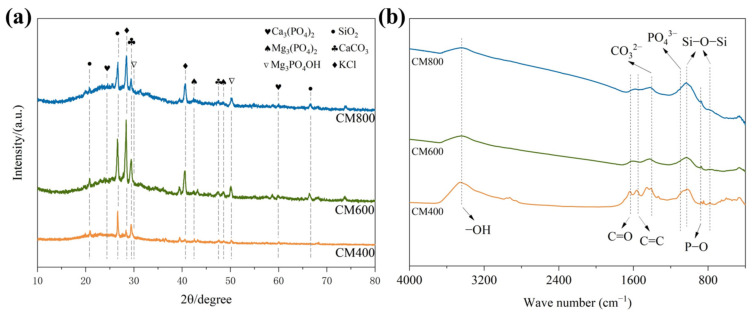
XRD and FTIR spectra of three kinds of CMBC. (**a**) XRD spectra and (**b**) FTIR spectra.

The XRD spectrum is depicted in Figure 2. The three kinds of CMBC exhibited the typical irregular wide peaks of biochar at approximately 24.3°. In addition to SiO_2_ (26.6°) (PDF#70–2517), the substances included a combination of embedded metal cations (Ca^2+^ and Mg^2+^) and anions (PO_4_^3–^ and CO_3_^2–^), such as Ca_3_(PO_4_)_2_ (PDF#09–0169), Mg_3_(PO_4_)_2_ (PDF#48–1167), and CaCO_3_ (PDF#70–0095) [31]. The higher the pyrolysis temperature, the more evident were the peaks of Ca_3_(PO_4_)_2_, Mg_3_(PO_4_)_2_, and Mg_3_PO_4_OH (PDF#47–0957), as the high-temperature pyrolysis was conducive to the formation of cationic and anionic conjugates, namely, CM400 was more likely to release inherent substances into solution. The peak of CaCO_3_ in CM800 decreased, which may be because high pyrolysis temperature (>600 °C) likely caused the decomposition of CaCO_3_ [32].

The FTIR spectrum was depicted in Figure 2. The –OH peak (3458 cm^−1^), the C=C stretching peaks (1566 cm^−1^ and 1455 cm^−1^), the C–O stretching vibration peak (1384 cm^−1^), and the C–O–C peak (876 cm^−1^) were observed [33]. Furthermore, the peak of CO_3_^2−^ and PO_4_^3−^ were located at 1415 cm^−1^ and 1096 cm^−1^ [34], as well as the peak of Si–O–Si at 1030 cm^−1^ and 780 cm^−1^ [35]. The results indicated that CO_3_^2−^, PO_4_^3−^, and SiO_2_ were retained in CMBC. As shown in the FTIR spectra (Figure 2b), compared with CM400, the peak value of functional groups such as –OH, C=O, and C=C of CM800 was weakened. Hence, as the pyrolysis temperature rose from 400 °C to 800 °C, there was a decrease in the functional groups. To sum up, CM400 solidified less inherent phosphorus, so more inherent phosphorus could be released into the solution to participate; at the same time, its functional groups was rich, which made the complexation stronger.

### 3.2. Pb(II) Adsorption

#### 3.2.1. Effect of pH

**Figure 3:**
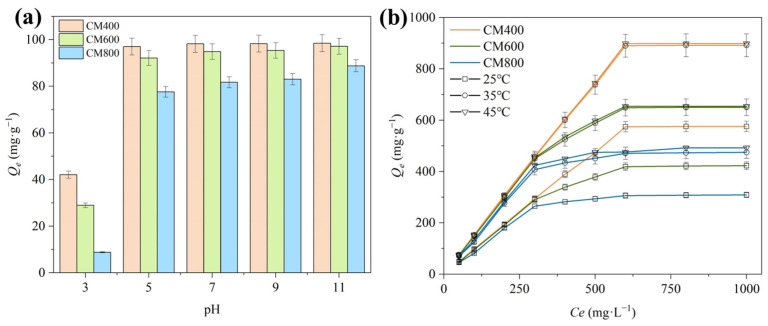
The effect of pH and temperature on the adsorption. (**a**) pH and (**b**) temperature. Experimental conditions: [initial concentration of Pb(II)] = 100 mg·L^−1^, [dosage] = 1 g·L^−1^.

The effect of pH on adsorption is shown in Figure 3. When pH = 3, *Q_e_* plunged (42.09, 28.95, and 8.77 mg·g^−1^ for CM400, CM600, and CM800, respectively). Although the strong acid condition promoted the release of PO_4_^3−^, which can form precipitation with Pb(II) [36], *Q_e_* of CM400 was only 42.09 mg·g^−1^. Therefore, H^+^ likely competed with Pb(II) for the adsorption sites, and the protonated surface functional groups also exhibited electrostatic repulsion [37]. Under alkaline conditions, there were more negative charges on the surface of CMBC and more free hydroxyl groups provided adsorption sites [38]. Besides, OH^−^ was involved in adsorption, such as Pb_10_(PO_4_)_6_(OH)_2_. The *Q_e_* of CM400 was almost unchanged from pH = 5 to pH = 11 (97.01 vs. 98.47 mg·g^–1^). This not only showed that CM400 functions well in a wide range of pH, but also showed that although the adsorption sites increased steadily, *Q_e_* did not vary because Pb(II) was adsorbed. In conclusion, the CMBC had the best adsorption effect for Pb(II) in an alkaline environment.

#### 3.2.2. Effect of Temperature

**Figure 4:**
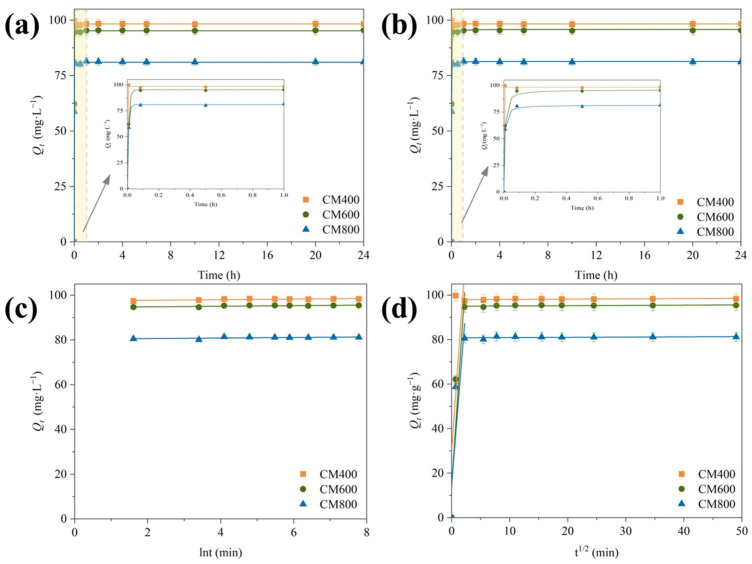
Adsorption kinetics of Pb(II) by CMBC. (**a**) Pseudo-first-order model, (**b**) pseudo-second-order model, (**c**) Elovich model, and (**d**) internal diffusion model. Experimental conditions: [initial concentration of Pb(II)] = 100 mg·L^−1^, [dosage] = 1 g·L^−1^, [temperature] = 25 °C.

The temperature had an impact on adsorption (Figure 3). The effect of pH on adsorption is shown in Figure 4. When the original concentration of Pb(II) was ≤300 mg·L^−1^, the removal rates were not affected by temperature (98.38% vs. 99.80% at 25 and 45 °C). Once the initial concentration was 500 mg·L^−1^ or 1000 mg·L^−1^, the adsorption was enhanced with temperature. For example, when the initial concentration was 1000 mg·L^−1^, *Q_e_* of CM600 was 422.59 mg·g^−1^ and that of CM800 was 308.63 mg·g^−1^ at 25 °C, but *Q_e_* of CM600 and CM800 were 425.1 and 320.1 mg·g^−1^ at 45 °C. Therefore, the adsorption by CMBC was endothermic, and CM600 and CM800 were more significantly affected by temperature.

#### 3.2.3. Adsorption Kinetics

The fitting results of CMBC are shown in Table S1 and Figure 4. The Elovich model and internal diffusion model could not fit the adsorption of Pb(II) well, for the R^2^ values of three kinds of CMBC were all less than 0.5. Thus, the surface adsorption energy was not uniformly distributed during adsorption, and the internal diffusion is not the sole rate-determining factor as C ≠ 0. For CM400 and CM600, the pseudo-second-order kinetic model fitted the adsorptions better (0.9997 and 0.9998 vs. 0.9999 and 0.9999), by contrast, for CM800, the pseudo-first-order kinetic model described the adsorption slightly more accurately (0.9998 vs. 0.9988). Therefore, physisorption and chemisorption likely coexisted during adsorption by CM400 and CM600, while physisorption dominated Pb(II) adsorption by CM800 [39]. Physisorption was reversible and slow [1], which can be verified by the lower *Q_e_* of CM800 than that of CM400 (98.21 vs. 81.28 mg·g^−1^). Compared with other research data, three kinds of CMBC were all effective Pb(II) adsorbents; among them, CM400 showed the best effect, as its *Q_e_* reached 691.34 mg·g^−1^ (Table 2).

#### 3.2.4. Isothermal Adsorption

**Figure 5:**
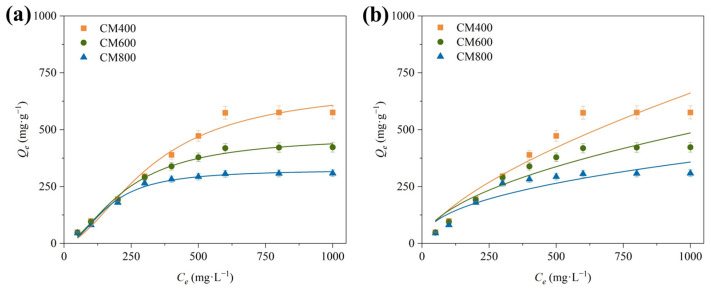
Isothermal adsorption model of Pb(II) by CMBC. (**a**) Langmuir model and (**b**) Freundlich model. Experimental conditions: [dosage] = 1 g·L^−1^, [temperature] = 25 ℃.

The isothermal adsorption model was presented in Figure 5. The R^2^ values of the Langmuir model were 0.9981, 0.9966, and 0.9945 for CM400, CM600, and CM800, respectively. Those of the Freundlich model were 0.9934, 0.9916, and 0.9899 for CM400, CM600, and CM800, respectively. Clearly, Langmuir model fit the adsorption of Pb(II) better. Therefore, the adsorption of Pb(II) by CMBC was monolayer adsorption [40].

**Table 2:** Adsorption capacities of different adsorbents for Pb(II).

Adsorbent	Biomass	Pyrolyzation (°C)	Balance Time (min)	*Q_max_* (mg·g^–1^)	Refs.
Nitrogen- and phosphorus-enriched biochar	*C. oleifera* shells	550	200	723.6	[41]
Biochar-supported phosphate-doped ferrihydrite	Corn stalks	400	360	247	[42]
Phosphorous-laden biochar	Sawdust	500	1440	568.22	[43]
Dairy manure-derived biochar	Cow manure	200	180	140.76	[44]
Biochar-supported nanoscale ferrous sulfide composite	Peanut shells	250	150	88.06	[45]
Sugarcane bagasse biochar	Sugarcane bagasse	600	360	2.5	[46]
Iron-sulfur co-doped biochar composite	rice straw	300	240	631.7	[3]
Cow manure biochar	Cow manure	400	0.5	691.34	This work
		600	5	473.36	
		800	5	323.83	

### 3.3. Inherent Phosphorus Release

Pyrolysis temperature was a decisive factor for morphology and crystal structure of phosphorus in biochar [47]. In water, *Qe*, the solution phosphorus content reached 0.620, 0.495, and 0.329 mg·g^−1^. The results were summarized in Figure S1, and as shown in Table S2, the R^2^ values of the pseudo-first-order model for phosphorus release by CM400, CM600, and CM800 were 0.9786, 0.9667, and 0.9296, respectively. Simultaneously, those of pseudo-second-order model were 0.9954, 0.9870, and 0.9786, respectively. Therefore, the phosphorus release by CMBC involved both physical and chemical reactions, which was consistent with the phosphorus release from reed straw biochar modified with phosphate [48].

During Pb(II) adsorption, the process of phosphorus release from CMBC was also affected by the pH and temperature (Figure S2). A higher reaction temperature promoted phosphorus release (0.166 mg·L^−1^ at 45 °C vs. 0.045 mg·L^−1^ at 25 °C), which inferred that phosphorus release was endothermic.

The higher the pH, the more of the phosphorus content remained (Figure S2). When pH = 3, the remaining phosphorus content was 0.047, 0.048, and 0.035 mg·L^−1^ for CM400, CM600, and CM800, respectively, while when pH = 11, they were 0.423, 0.274, and 0.197 mg·L^−1^ for CM400, CM600, and CM800, respectively. Under the initial alkaline reaction conditions, phosphorus mostly existed as HPO_4_^2−^ and PO_4_^3−^, whereas phosphorus mostly existed in the form of HPO_4_^2−^ and H_2_PO_4_^−^ under neutral conditions [49]. Strong acidic conditions promoted the release of PO_4_^3−^ from CMBC, while in an alkaline environment, OH^−^ also precipitated with Pb(II) [50]. Thus, the amount of phosphorus consumed for precipitation was reduced, contributing to a higher phosphorus content at pH = 11.

Considering the adsorption capacity mentioned above, the adsorption capacity significantly exceeded that at pH = 3 and the residual phosphorus content at pH = 11 considerably exceeded that at pH = 3. Thus, under alkaline conditions, the chemical precipitation of phosphate likely contributed less toward adsorption, whereas complexation of functional groups and Pb(II) had a considerably greater contribution. 

**Figure 6:**
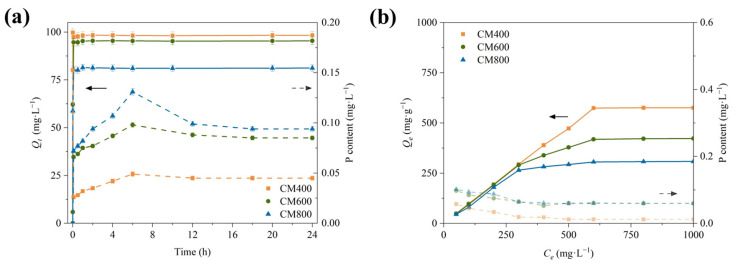
The relationship between the residual phosphorus content and *Q_t_*. (**a**) the relationship between the residual phosphorus content and *Q_t_* at different reaction times [initial concentration of Pb(II)] = 100 mg·L^−1^, [dosage] = 1 g·L^−1^, [temperature] = 25 °C, (**b**) The relationship between the residual phosphorus content and *Q_t_* in reaction with different initial concentrations, [reaction times] = 24 h, [dosage] = 1 g·L^−1^, [temperature] = 25 °C.

Excluding the pH and temperature, the Pb(II) concentration also affected the phosphorus release (Figure 6). For example, when the initial concentration of the Pb(II) solution was 100 mg·L^−1^, *Q_e_* of CM400 was more than that of CM800 (98.38 vs. 81.19 mg·g^−1^), however, the residual phosphorus content of CM400 was less than that of CM800 (0.05 vs. 0.09 mg·L^−1^). Thus, when Pb(II) was less than 100 mg·L^−1^, the main adsorption mechanism likely was chemical precipitation, while the main mechanisms transformed into ion exchange and complexation. When Pb(II) solution was 100 mg·L^−1^, the residual phosphorus content increased rapidly within 6 h and then decreased. Finally, the residual phosphorus content remained stable 12 h after the onset of adsorption. Because the solution became alkaline when CMBC was placed in, the inherent phosphorus was released slowly at first. Further, Pb(II) was adsorbed by electronegative CMBC via electrostatic attraction, and underwent a complexation reaction and ion exchange with the functional groups. During the adsorption, OH^−^ was consumed, and the reaction solution tended to be neutral. Thus, the phosphorus release accelerated, and the remaining content remained stable.

### 3.4. Adsorption Mechanism Analysis

**Figure 7:**
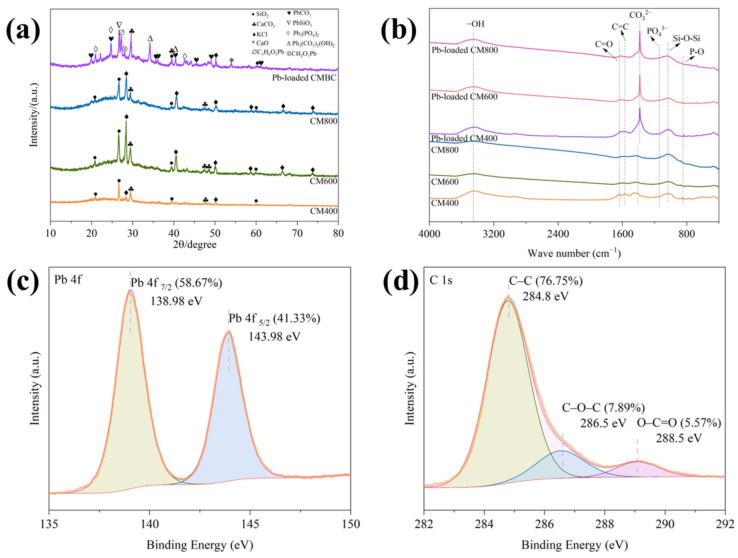
XRD, FTIR and XPS spectra of Pb(II)–loaded ((**a**): XRD spectrum, (**b**): FTIR spectrum, (**c**, **d**): XPS spectra).

The XRD of Pb(II)-loaded CMBC is shown in Figure 7. The characteristic peaks at 20.8°, 27.28°, and 42° represented Pb_3_(PO_4_)_2_ (PDF#73–0834), which confirm that inherent phosphorus released and reacted [42]. The characteristic peak at 26.6° and 40° represented PbSiO_3_ (PDF#74–1101) and PbCO_3_ (PDF#85–1088), indicating that CMBC adsorbed Pb(II) via precipitation and ion exchange [51]. The precipitation equilibrium constant (Ksp) of PbCO_3_ was 3.3 × 10^−14^, while those of CaCO_3_ and MgCO_3_ were 2.8 × 10^−9^ and 2.6 × 10^−5^. Therefore, a few Pb ions were exchanged with the precipitates. Moreover, the peak at 27.4° represented C_3_H_6_O_3_Pb, and the peak at 27.8° represented CH_2_O_3_Pb (PDF#14–0831), indicating an ion exchange [52]. There were Pb_3_(CO_3_)_2_(OH)_2_ (PDF#72–1144) and Pb_10_(PO_4_)_6_(OH)_2_ (PDF#87–2478), verifying that the hydroxyl group participated in adsorption [53].

The FTIR of Pb(II)-loaded CMBC is shown in Figure 7. The –OH group peak shifted, presumably because the hydroxyl group complexed with Pb(II). The C=O vibration peak of the carboxyl and carbonyl groups at 1640 cm^−1^ disappeared, possibly because the oxygen–containing functional group ligand complexed with Pb(II) [54]. The peak value of Si–O–Si was positively correlated with the adsorption, indicating that the cation–π bond interaction existed, because positron coordination improved the adsorption if the peak value positively correlated with the adsorption [55]. The C–O–C peak at 876 cm^−1^ shifted, indicating that H^+^ provided by the carboxyl and hydroxyl groups exchanged with Pb(II) [56].

The XPS of Pb(II)-loaded CMBC were depicted in Figure 7. The peak of Pb 4f (138 eV) appeared after the adsorption and the valence of Pb(II) did not change, so there were no oxidation-reduction reactions that occurred in Pb(II). The characteristic peaks of C 1s at 284.80 eV, 286.35 eV, 288.60 eV, and 285.35 eV represented C–C, C–O, COOH, and C–OH groups, respectively [48], and the surface functional groups were crucial to adsorption.

**Figure 8:**
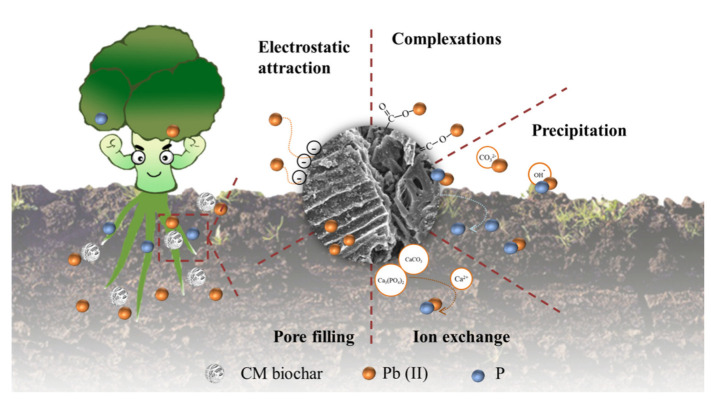
Surface interaction between Pb(II) and CM functional materials.

CMBC adsorbed Pb(II) in the solution rapidly and efficiently. The adsorption mechanisms include physical adsorption, ion exchange, cation–π bond interaction, chemical precipitation, electrostatic attraction, and surface complexation (Figure 8). Pb(II) was adsorbed via electrostatic attraction because CMBC was electronegative, and the ion diameter of Pb(II) (0.12 nm) was adsorbed via pore filling. The variation in Si–O–Si peak after adsorption was positively correlated with the Pb(II) adsorption, thus, CMBC adsorbed Pb(II) via cation–π bond. Pb(II) was adsorbed onto the biochar surface via ion exchange, replacing H^+^ from the basic functional groups. The inherent phosphorus reacted with Pb(II) to form precipitates, and the oxygen-containing functional group ligands on the surface of tCMBC were complexed with the Pb(II). 

The adsorption mechanisms differed under diverse reaction conditions. Under alkaline conditions, the complexation had a greater contribution than precipitation, and the main adsorption mechanisms transformed from chemical precipitation to ion exchange and complexation.

The main adsorption mechanisms for CM400 were chemical precipitation, surface complexation, and ion exchange. By contrast, CM800 had a rich pore structure and a large pore size; hence, its physical adsorption ability was strong.

### 3.5. Analysis of Pot Experiment Results

The effects of CMBC on soil decontamination and fertilizer improvement were also investigated, and the contents are shown in Figure S3. On the third day after sowing, for T_1_, T_2_, T_3_, and CK_1_, three, three, two, and three buds were noted, however, none of the CK_2_ groups sprouted at that time. Application of CMBC can reduce the germination inhibition of Chinese cabbage growing in lead-contaminated soil. It was speculated that CMBC can reduce the biotoxicity of Pb(II) to Chinese cabbage growth by solidifying soil heavy metals during the germination stage of Chinese cabbage. After the Chinese cabbages ripened, the roots, stems, and leaves were cut for digestion and detection. For CK_2_, the Pb(II) content in leaves was 0.04 mg·L^−1^, while it was 0.03, 0.02, and 0.00 mg·L^−1^ for T_1_, T_2_, and T_3_, a reduction of 26.82%, 40.50%, and 99.72%, respectively. The Pb(II) content in stems was 0.04 mg·L^−1^ for CK_2_, while it was 0.03, 0.02, and 0.01 mg·L^−1^ for T_1_, T_2_, and T_3_. Furthermore, the Pb(II) content in roots for CK_2_ was 0.86 mg·L^−1^, while it was 0.06, 0.07, and 0.04 mg·L^−1^ for T_1_, T_2_, and T_3_, a reduction of 92.57%, 91.48%, and 94.92%, respectively. After the Chinese cabbage planted in lead-contaminated soil ripened, by comparing the data of CK_2_, T_1_, T_2_, and T_3_, it was found that the Pb(II) content in three parts of the Chinese cabbage in the four groups was different. When biochar was applied, the Pb(II) content in the cabbage decreased. The cabbage treated with CM800 (T_3_) had the least Pb(II) content. In other words, if biochar was applied to lead-contaminated soil and regardless of the type of biochar applied, both had an effect on the Pb(II) content in Chinese cabbage. The application of CMBC to lead-contaminated soil can achieve decontamination, and CM800 was most effective. Therefore, it is speculated that CMBC can reduce the bioenrichment of Pb(II) in Chinese cabbage by solidifying soil heavy metals during the growth stage of Chinese cabbage. In short, the biochar likely immobilized Pb(II) in the soil to reduce the amount of plant uptake [57].

The phosphorus content in leaves was 3.85, 1.96, 1.88, 6.65, and 1.41 mg·L^−1^ for T_1_, T_2_, T_3_, CK_1_, and CK_2_, respectively. The phosphorus content in stems was 1.85, 1.88, 1.37, 2.27, and 0.41 mg·L^−1^. The phosphorus content in roots was 1.83, 1.65, 1.14, 2.39, and 0.62 mg·L^−1^, respectively. Compared with CK_2_, application of CMBC increased phosphorus in plants. In summary, the application of CMBC adsorbed Pb(II) in soil, reduced the Pb(II) content in crops, and promoted plant growth effectively.

## 4. Conclusions

CM400 proved to be an effective adsorbent; its *Q_e_* reached 691.34 mg·g^−1^, and it rapidly adsorbed 98.36 mg·g^−1^ of Pb(II) within 30 s. The adsorption mechanisms of Pb(II) by CMBC include ion exchange, physical adsorption, electrostatic attraction, chemical precipitation, surface complexation, and cation–π bond interaction. CMBC released phosphorus and the phosphorus released from CM400 reached 0.620 mg·g^−1^. The phosphorus release was endothermic, and the higher the pH, the more the phosphorus content remained. Based on the residual phosphorus content and adsorption effect, complexation rather than chemical precipitation had a greater contribution toward adsorption. Besides, as the concentration of Pb(II) increased, the main adsorption mechanisms likely transformed from chemical precipitation to ion exchange and complexation. CMBC not only had a good effect on Pb(II) removal in the solution, but also immobilized the Pb(II) in the soil to restrain the plant uptake and promoted plant growth.

## Figures and Tables

**Table 1:** Basic elemental composition, specific surface area, and pore structure of CMBC.

	CM400	CM600	CM800
C (%)	28.11	37.14	39.21
H (%)	5.05	2.53	1.63
O (%)	41.18	40.34	37.89
P (%)	0.61	0.34	0.27
O/C	1.48	1.09	0.97
H/C	0.18	0.07	0.04
BET (m^2^·g^−1^)	2.51	11.78	12.93
Vtotal (m^3^·g^−1^)	0.05	0.03	0.03
Average pore size (Å)	837.94	90.31	108.91

## Data Availability

Not applicable.

## Supplementary Materials

The following supporting information can be downloaded at: https://www.mdpi.com/article/10.3390/toxics11010001/s1, Figure S1: Phosphorus release kinetics in deionized water; Figure S2: The effect of pH (a) and temperature (b) on phosphorus release; Figure S3: Pb(II) and phosphorus contents in each part of the crops; T1: 1 g CM400 application, T2: 1 g CM600 application, T3: 1 g CM800 application, CK1: no pollution treatment, CK2: no adsorbent application. Table S1: Table of fitting parameters of kinetic model and isotherm model of Pb(II) absorbtion. Table S2: Table of fitting parameters of kinetic model, Elovich model, and Internal diffusion model of phosphorus release.
