# Supplementary material for: Rapid and Effective Lead Elimination Using Cow Manure Derived Biochar: Balance between Inherent Phosphorus Release and Pollutants Immobilization
Source: Toxics. 2022 Dec 20;11(1):1. doi: 10.3390/toxics11010001 (PMC9861172; doi:10.3390/toxics11010001)
Supplement: Supplementary file 1 [file toxics-11-00001-s001.zip › toxics-2077852-supplementary.pdf]

Supplementary materials

Rapid and Effective Lead Elimination Using Cow Manure Derived Biochar: Balance between Inherent Phosphorus Release and Pollutants Immobilization

Huabin Wang [1,2], Yi Wen [1,2], Yu Ding [3], Zhiqiang Yue [4], Dan Xu [3], Ying Liu [1,2], Yong Zhang [1,2], Rui Xu [1,2,*] and Weiqing Zeng [1,4,*]

1 School of Energy and Environment Science, Yunnan Normal University, Kunming 650500, China; hbwang@ynnu.edu.cn (H.W.); wyaquarius@foxmail.com (Y.W.); Liuying13769153740@163.com (Y.L.); yongzhang7805@126.com (Y.Z.)

2 Yunnan Key Laboratory of Rural Energy Engineering, Kunming 650500, China

3 Baoshan City Longyang Rural Energy Workstation, Baoshan 678000, China; dingyudoug1130@163.com (Y.D.); 2003xudan@163.com (D.X.)

4 Yuxi Agricultural Environmental Protection and Rural Energy Workstation, Yuxi 653100, China; tdww803@163.com

* Correspondence: ecowatch_xr@163.com (R.X.); zdfad707@163.com (W.Z.); Tel.: +86-27-87792151 (R.X.); Fax: +86-27-87792151(R.X.)

Text S1

$$Q_e = (C_0 - C_e) \times \frac{V_0}{m} \quad \text{(S1)}$$

$$Q_t = Q_e(1 - e^{-k_1 t}) \quad \text{(S2)}$$

$$\frac{t}{Q_t} = \frac{1}{k_2 Q_e^2} + \frac{t}{Q_e} \quad \text{(S3)}$$

$$Q_t = a + b\ln(t) \quad \text{(S4)}$$

$$Q_t = K_{id} t^{0.5} + C \quad \text{(S5)}$$

$$Q_e = \frac{Q_m C_e K_L}{1 + K_L C_e} \quad \text{(S6)}$$

$$Q_e = K_F C_e^{\frac{1}{n}} \quad \text{(S7)}$$

In the equations, Q_e represents the adsorption capacity in mg·g^{-1}; C_0 and C_e represent the mass concentration of Pb(II) at the initial and adsorption equilibrium in mg·g^{-1}, respectively; V_0 is the volume of the Pb(II) solution (L); and m represents the dosage of biochar in g; Q_t represents the adsorption capacity at time t in mg·L^{-1}; Q_e represents the equilibrium adsorption capacity in mg·g^{-1}; k_1 represents the reaction rate constant of the pseudo first–order kinetic equation in min^{-1}; k_2 is the reaction rate constant of the pseudo second–order kinetic equation in g·(mg·min)$^{-1}$; and a and b are the initial adsorption rate and activation energy–related constants in mg·(g·min)$^{-1}$ and g·mg^{-1}, respectively. Further, K_{id} is the intragranular diffusion rate constant in mg·(g·min$^{1/2}$)$^{-1}$, C is the boundary layer effect, C_e represents the mass concentration of Pb(II) in mg·L^{-1}, Q_m represents the theoretical maximum adsorption capacity in mg·g^{-1}, K_L represents the equilibrium constant of the Langmuir equation, K_F is the Freundlich constant, and n is the empirical constant.

Table S1. Table of fitting parameters of kinetic model and isotherm model of Pb(II) absorbtion.

Models	Parameters	CM400	CM600	CM800
Pseudo-first-order kinetic model	Q_e (mg·g^{-1})	98.21	95.3	81.14
	k_1	10.85	9.8	8.68
	R^2	0.9997	0.9998	0.9998
Pseudo-second-order kinetic model	Q_e (mg·g^{-1})	98.36	95.46	81.28
	K_2	2.96	2.41	2.15
	R^2	0.9999	0.9999	0.9988
Elovich model	A (mg·g^{-1}·min^{-1})	97.45	94.55	80.39
	B (g·mg^{-1})	0.14	0.13	0.12
	R^2	0.5552	0.5331	0.2222
Internal diffusion model	K_{id1} mg·(g·min$^{1/2}$)$^{-1}$	36.33	38.97	32.53
	C (mg·g^{-1})	30.09	14.04	14.53
	R^2	0.0636	0.7144	0.5924
	K_{id2} mg (g·min$^{1/2}$)$^{-1}$	0.01	0.01	0.01
	C (mg·g^{-1})	97.93	94.99	80.81
	R^2	0.1922	0.2234	0.0426
Langmuir isotherm model	Q_e (mg·g^{-1})	691.34	473.36	323.83
	K_L	3.47	8.79	3.25
	R^2	0.9981	0.9966	0.9945
Freundlich isotherm model	n	1.54	1.90	2.30
	K_F	7.51	12.90	17.71
	R^2	0.9934	0.9916	0.9899

Table S2. Table of fitting parameters of kinetic model, Elovich model, and Internal diffusion model of phosphorus release.

Models	Parameters	CM400	CM600	CM800
Pseudo-first-order kinetic model	Q_e (mg·g^{-1})	0.6086	0.4832	0.3166
	k_1	24.50	21.77	16.89
	R^2	0.9786	0.9667	0.9296
Pseudo-second-order kinetic model	Q_e (mg·g^{-1})	0.6149	0.4897	0.3241
	K_2	104.05	98.98	83.51
	R^2	0.9954	0.9870	0.9786
Elovich model	a (mg·g^{-1}·min^{-1})	0.52	0.40	0.23
	b (g·mg^{-1})	0.02	0.02	0.02
	R^2	0.5168	0.5168	0.5113
Internal diffusion model	K_{id1} mg·(g·min$^{1/2}$)$^{-1}$	0.0064	0.0018	0.0087
	C (mg·g^{-1})	0.5027	0.3992	0.2021
	R^2	0.4009	0.9606	0.3945
	K_{id2} mg (g·min$^{1/2}$)$^{-1}$	9.7950	0.0010	0.0012
	C (mg·g^{-1})	0.5882	0.4610	0.2903
	R^2	0.0355	0.0509	0.0872

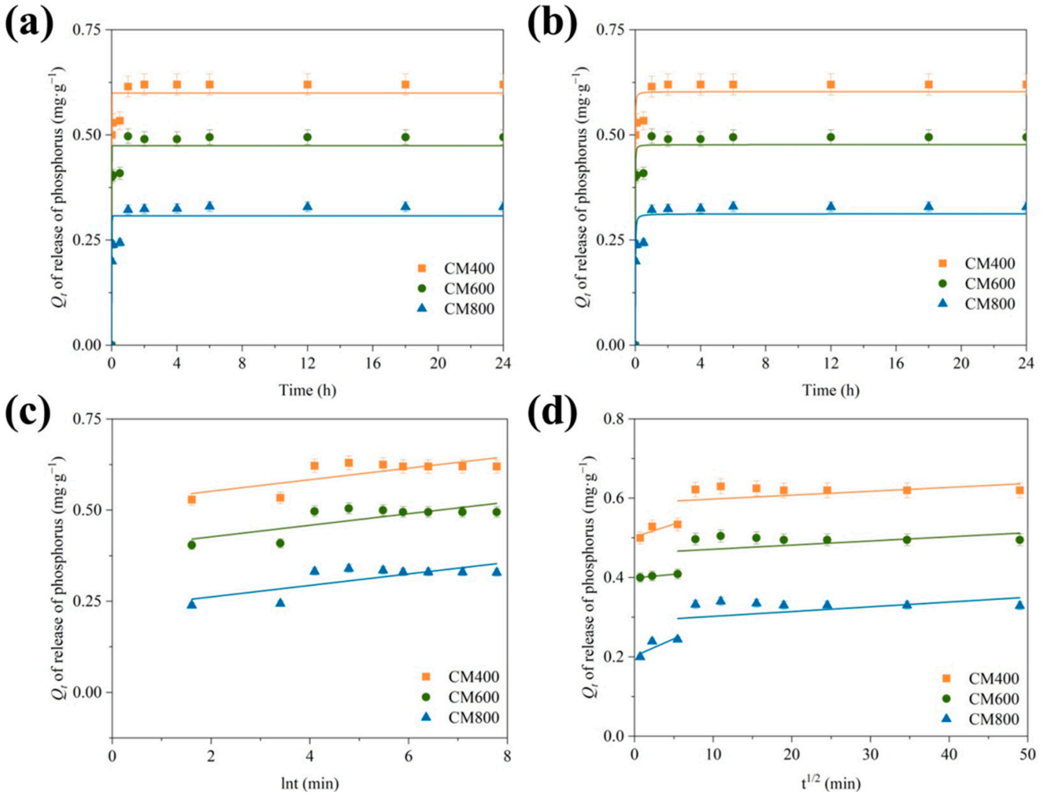

Figure S1. Phosphorus release kinetics in deionized water. Experiment condition: [dosage] = 1 g·L^{-1}, [temperature] = 25 °C.

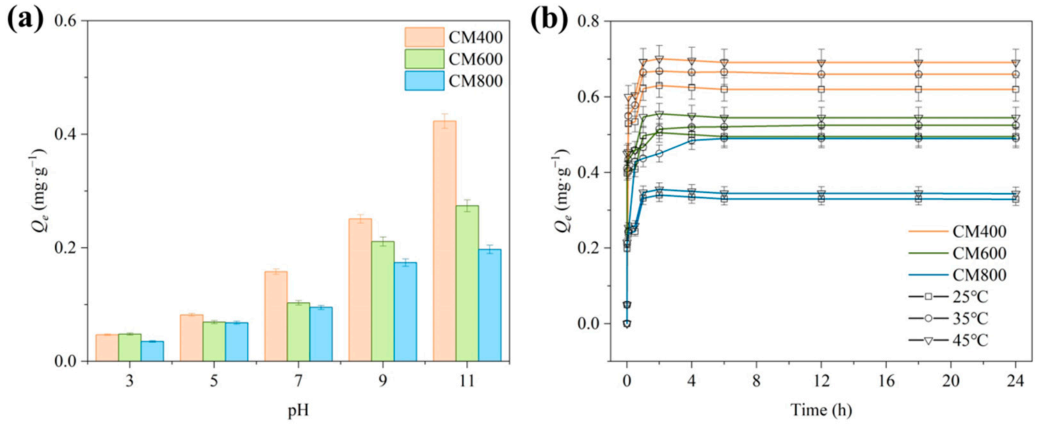

Figure S2. The effect of pH (a) and temperature (b) on phosphorus release. Experiment condition: [dosage] = 1 g·L^{-1}.

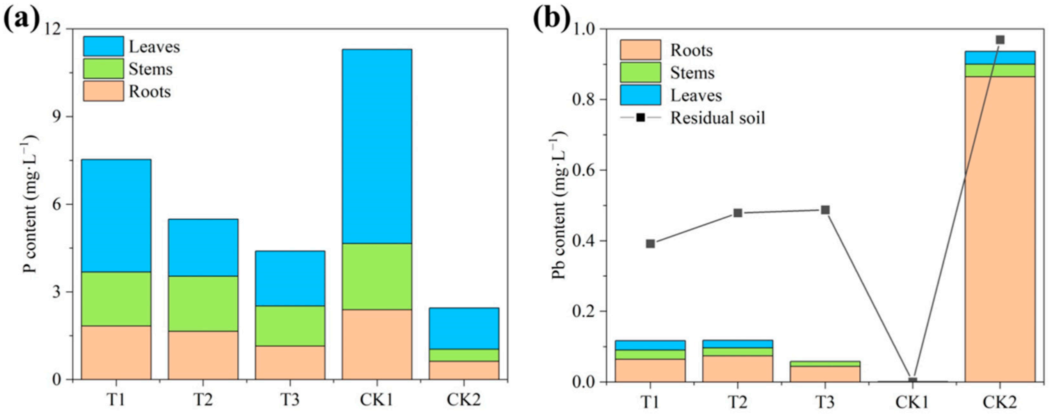

Figure S3. Pb(II) and phosphorus contents in each part of the crops (a: phosphorus contents, b: Pb(II) contents). T1: 1 g CM400 application, T2: 1 g CM600 application, T3: 1 g CM800 application, CK1: no pollution treatment, CK2: no adsorbent application.
